# A rare case of mesenteric vessel thrombosis post caesarian section—An underdiagnosed entity

**DOI:** 10.1016/j.ijscr.2020.02.029

**Published:** 2020-02-19

**Authors:** Richa Pawar, Komal Brar, Chanchal Malhotra, Sonia Chhabra, Deepshikha Rana, Anubha Gupta

**Affiliations:** a Department of Pathology, Pt. B D Sharma PGIMS, Rohtak, India; b Department of Oncosurgery, Bhagwan Mahavir Cancer Hospital, Jaipur, India

**Keywords:** Thrombosis, Mesenteric vessel, Postpartum, Thrombophilia, Hypercoagulable, Case report

## Abstract

**Introduction:**

Respiratory distress is an uncommon clinical event after caesarean section and occurs due to pulmonary thromboembolism. Various causes of pulmonary thromboembolism are thrombophlebitis, ovarian venous thrombosis and mesenteric vein thrombosis.

**Presentation of case:**

We report a case of 30 year old female who presented with respiratory distress after eight days of uneventful caesarian section. On emergency explorative laparotomy, small gut was found to be gangrenous, so resection of the segment was performed. On histopathological examination, there was ischaemic necrosis of bowel with presence of large thrombus in mesenteric vessel. On correlating radiological findings of pulmonary thromboembolism and mesenteric vessel thrombosis with bad obstetric history, a possibility of Antiphospholipid syndrome (APS) was suggested in this case. Unfortunately, patient died the day following laparotomy so there was insufficient time to evaluate the patient for thrombophilic disorders.

**Discussion:**

Pregnancy and perpeurium are associated with higher risk of thrombosis as these are hypercoagulable states. Operative delivery and history of thrombophilia in previous pregnancies (APS) are other predisposing factors which lead to increased thrombotic state and pulmonary thromboembolism.

**Conclusion:**

High index of suspicion for thrombophilic disorders is required in postpartum patients presenting with respiratory distress as prompt diagnosis and urgent intervention can save patient’s life.

## Highlights

• Mesenteric vessel thrombosis is a rare event after caesarean section.
• It can lead to pulmonary thromboembolism (PTE) and respiratory distress.
• Pregnancy, itself is a hypercoagulable state.
• Postpartum patients with bad obstetric history, respiratory distress must be evaluated for thrombophilic disorders.
• High index of suspicion is required for thrombophilic disorders.
• Prompt diagnosis and urgent intervention can save patient’s life.

## 1 Introduction

Respiratory distress is an uncommon clinical event after caesarean section and occurs due to pulmonary thromboembolism. Various causes of pulmonary thromboembolism include thrombophlebitis, ovarian venous thrombosis and mesenteric ischemia. Mesenteric vein thrombosis is a well-known cause of mesenteric ischemia [1]. Pregnancy and perpeurium are associated with shift of coagulation and fibrinolytic system towards hypercoagulability to reduce risk of bleeding during delivery, but this can result in higher risk of thrombosis. Apart from pregnancy, advanced maternal age, higher parity, operative delivery, obesity, immobilization, heart disease, history of thrombosis and thrombophilia are other predisposing factors [2,3]. We report a case of 30 year old female who presented with respiratory distress on 8th postoperative day following uneventful caesarian section. Unfortunately, patient died the day after laparotomy leaving behind the unsolved mystery that led to catastrophic events.

## 2 Case report

We report the case of a mesenteric vessel thrombosis in a 30 year old female, G5 P3, eight days after caesarean section. She also had history of two spontaneous abortions between P2 and P3. She had undergone uneventful caesarean section in view of fetal distress with meconium stained liquor, delivered a healthy female baby and was discharged on fifth post-operative day. Patient developed fever with rigors & chills and respiratory distress on sixth post-operative day. Also had repeated episodes of vomiting which were non-bilious, non-projectile in nature. Patient had normal bowel and bladder habits till 8th post-operative day.

Eight days after her caesarean section, she presented to emergency department with respiratory distress. On examination, she was afebrile and her abdomen was soft. On auscultation there was decreased air entry in bilateral lungs. Her blood pressure on arrival was 156/110 mm of Hg and pulse 102/min. Patient was nebulized and put on antibiotics, oral Labetalol 20 mg, injection Amlodipine10 mg stat, injection MgSO_4_ loading followed by maintenance. Vital charting was done; patient had persistent tachycardia during next 4 days. All baseline investigations were sent. Subsequently, on Day 5 of admission patient developed abdominal distension with absence of bowel sounds and also complained of numbness in left arm. On examination of pulses: right sided radial artery volume was good but low volume was noticed in left sided radial, brachial, bilateral dorsalis paedis and posterior tibial artery. Right arm B.P – 128/70 mm of Hg, Left arm B.P – 80/60 mm of Hg. Abdomen was diffusely tender. Probable diagnosis of sepsis with intestinal obstruction was made.

**Fig. 1:**
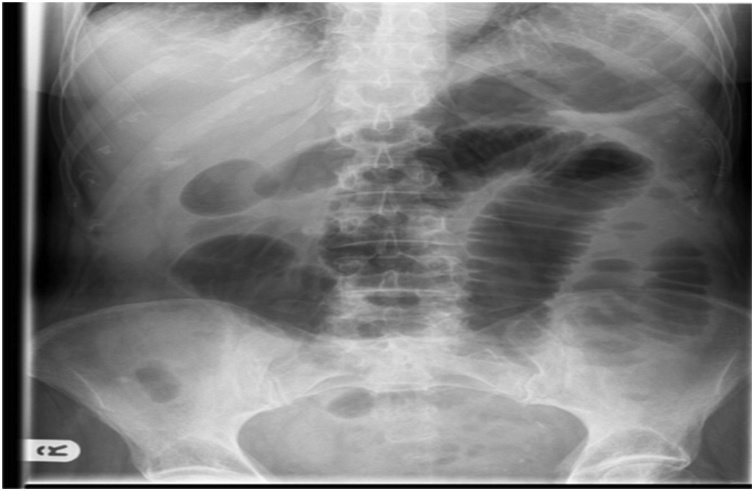
X-ray of abdomen show multiple dilated small gut loops in central part of abdomen.

**Fig. 2:**
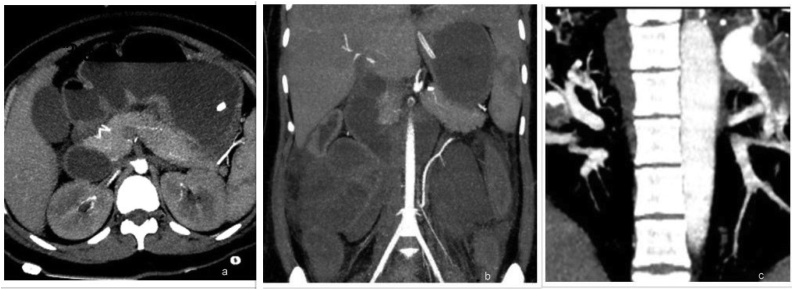
CT Angiography arterial phase images show: Completely occlusive non enhancing thrombus in superior mesenteric artery beginning at origin from aorta (Fig. 2a & b). Hypodense thrombus in Main pulmonary artery, Left and right pulmonary arteries and their branches (Fig. 2c).

Investigations: (Table 1)^#^ Hb 10.0 g/dL, platelets 2.8 lakh/mm^3^, total leucocyte count 18,500/mm^3^ with neutrophilia, serum creatinine 1.3 mg/dL, serum uric acid 7.7 mg/dL, electrocardiogram normal, chest X-ray- prominent vascular markings, abdominal X-Ray - dilated bowel loops (Fig. 1). USG whole abdomen showed prominent bowel loops and sluggish peristalsis. Color Doppler – No significant vascular abnormality, no obvious thrombosis/stenosis noted. CT – Angiography revealed complete occlusion of superior mesenteric artery (Fig. 2a and b), and saddle shaped thrombus in right and left pulmonary artery extending into bilateral upper and lower lobe segmental branches suggestive of pulmonary thromboembolism (Fig. 2c). CECT abdomen showed dilation of jejunal and ileal loops, likely small bowel ischemia.

**Table 1:** Table of investigations post admission (day wise).

Parameter	Day2	Day3	Day4
Hb (g/dL)	10.2	10.0	**9.5**
TLC (/cumm)	**17000**	**18000**	**18500**
Platelets (×10^9^/L)	260	280	250
aDLC	**70**/26/2/2	**76**/22/1/1	**78**/19/3
bPBF	N/N	N/N	N/N
RBS (mg/dL)	119	142	144
B.Urea (mg/dL)	32	44	**89**
S.Creatinine (mg/dL)	1.1	1.3	1.2
S.Uric acid (mg/dL)	5.6	5.5	**7.7**
S.AST (IU/L)	–	32	24
S.ALT (IU/L)	–	15	18
S.AlkPo_4_ (IU/L)	–	111	110
S.Bilirubin (mg/dL)	–	0.7	0.5
S.Albumin (g/dL)	–	3.3	3.0
A:G	–	1.0	0.8
S.Na^+^ (meq/L)	141	137	149
S.K^+^ (meq/L)	4.3	4.6	4.0
S.Ca^2+^ (mg/dL)	–	9.6	9.5
S.PO_4_ (mg/dL)	–	3.6	3.5

a (DLC = Differential leucocyte count indicates % of Neutrophils/Lymphocytes/Monocytes/Eosinophils).

b (PBF = Peripheral blood film; N/N = Normocytic normochromic picture).

Emergency exploratory laparotomy was performed with resection of gangrenous bowel with end to end anastomosis. Unfortunately, patient did not recover from shock and died on post-operative day 1 after laparotomy.

**Fig. 3:**
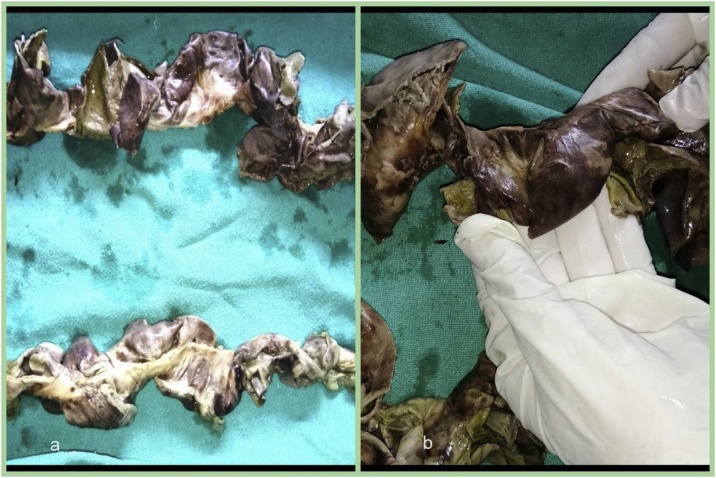
Gross specimen of gut showing gangrenous bowel comprising of jejunum and ileum with marked blackened and thinned out areas in bowel wall.

**Fig. 4:**
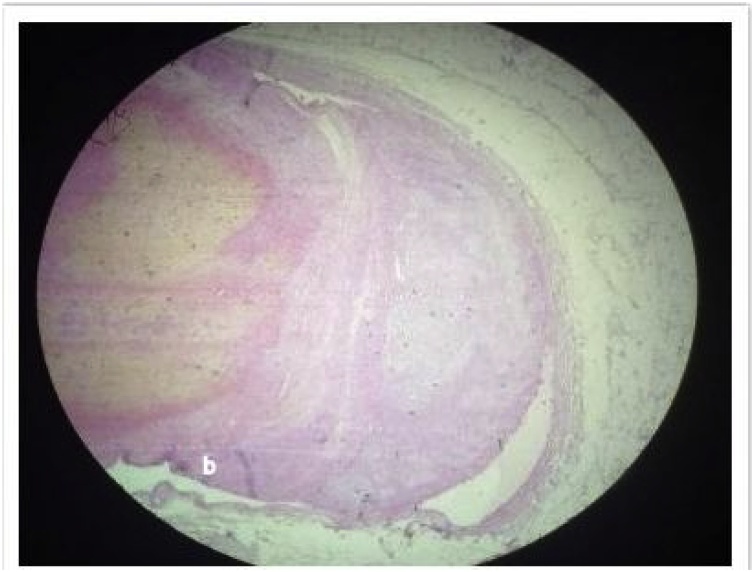
Photomicrograph show a fresh thrombus of 24 hour duration in mesenteric vein occluding upto 80 % of lumen.

Gangrenous segment of bowel was received in pathology department. Specimen was 60 cm in length and included jejunum and ileum. Grossly there was marked blackening and thinning out of areas in bowel wall (Fig. 3a and b). Microscopic sections examined showed features of mesenteric vessel thrombosis leading to infarction of segment of gut. Changes were typical of ischaemic damage comprising of necrosis and sloughing of mucosa, marked edema and haemorrhage in interstitium. Cut ends were viable. Sections examined from mesenteric vessels traced from the specimen show a fresh thrombus of 24 hour duration in mesenteric vein occluding upto 80 % of lumen (Fig. 4). This case is reported in line with the SCARE criteria [4].

## 3 Discussion

Acute mesenteric ischemia is a dreaded complication, which can be due to arterial occlusion by embolism or by thrombosis, venous thrombosis and non-occlusive mesenteric ischaemia. Outcome usually depends upon prompt diagnosis and is usually found to be worst in arterial thrombosis followed by non occlusive and mesenteric embolism. Acute mesenteric ischemia due to arterial embolism can be caused by cardiac diseases like arrthymia, valvular disease and myocardial infarction. It can also occur due to vasculitis, thrombosis or atheromatous disease [5]. Venous thromboembolism is a rare event during pregnancy. Pregnancy and perpeurium are themselves a state of hypercoagulability. Coagulopathies increase the risk of post-partum thromboembolic events by eightfold. In this case, pregnancy and C-section are contributing factors in causing mesenteric vessel thrombosis.

Considering the obstetric history of two spontaneous abortions in first trimester between P2 and P3, raise the suspicion of thrombophilic disorder. But unfortunately, patient expired and we did not get enough time to investigate the patient for deficiency of Protein C, Protein S and ATIII. On correlating radiological findings of pulmonary thromboembolism and mesenteric vessel thrombosis with bad obstetric history, a possibility of Antiphospholipid syndrome (APS) is strongly suggested in this case.

Antiphospholipid syndrome (APS) is an acquired thrombophilic disorder which causes activation of endothelial cells, monocytes and platelets resulting in an increased production of tissue factor and thromboxane A2 alongwith complement system activation. These factors along with typical changes in hemostatic system during normal pregnancy result in a hypercoagulable state which is responsible for thrombosis, the root cause for complications associated with Antiphospholipid syndrome (APS). Obstetric complications are the hallmark of antiphospholipid syndrome and include recurrent miscarriage, early delivery, prematurity, intrauterine growth restriction, fetal thrombosis, preeclampsia/eclampsia, HELLP syndrome, arterial or venous thrombosis and placental insufficiency. Systemic thromboembolic events in patients with APS are deep venous thrombosis (DVT), pulmonary embolism, stroke and transient ischaemic attacks [6,7].

A normal abdomino-pelvic ultrasound scan in a case of acute postpartum abdominal pain should be followed by a CT of the abdomen and pelvis. An angioscan is the gold standard to diagnose this pathology. Color Doppler ultrasonography is a noninvasive and useful modality for the diagnosis of PVT with sensitivity and specificity ranging from 66 % to 100 % [5,8].

In a stable patient, without signs of peritonitis and small thrombus burden, thrombolysis therapy can be started. I.V. administration of heparin is drug of choice for acute management and oral anticoagulation with warfarin for the patient whose clot appears stable. In a patient with peritonitis, superior mesenteric artery flow restoration and resection of non-viable bowel is mainstay of treatment. Embolectomy is done in such cases [9,10].

## 4 Conclusion

Postpartum thromboembolism is a rare phenomenon and is difficult to diagnose because of normal lab findings. CT angiography and CECT should be done in postpartum patients presenting with respiratory distress. A high index of suspicion is required for early diagnosis and prompt treatment to improve maternal outcome. Venous thromboembolism risk assessment should be performed and repeated in every pregnant woman by health care providers to reduce maternal mortality.

## Funding

Nil.

## Ethical approval

No research ethics approval was necessary for this case report. Written informed consent was obtained from the kin of patient for publication of this Case report and any accompanying images. A copy of the written consent is available for review by the Editor-in-Chief of this journal.

## Consent

Written informed consent was obtained from the kin of patient for publication of this case report and accompanying images. A copy of the written consent is available for review by the Editor-in-chief of this journal on request.

## Author contribution

Study concept: Dr. Richa Pawar, Dr. Sonia Chhabra.

Writing the paper: Dr. Komal Brar, Dr. Anubha Gupta.

Data Interpretation: Dr. Chanchal Malhotra, Dr. Deepshikha Rana.

## Registration of research studies

Not applicable.

## Guarantor

Dr. Komal Brar.

## Provenance and peer review

Not commissioned, externally peer-reviewed.

## Declaration of Competing Interest

We have no conflict of interest.
